# Amplified fragment length polymorphism analysis supports the valid separate species status of *Lucilia caesar* and *L. illustris* (Diptera: Calliphoridae)

**DOI:** 10.1080/20961790.2017.1398286

**Published:** 2017-12-08

**Authors:** Christine J. Picard, Jeffrey D. Wells, Anne Ullyot, Knut Rognes

**Affiliations:** a Department of Biology & Forensic and Investigative Sciences Program, Indiana University-Purdue University Indianapolis, Indianapolis, IN, USA; b Department of Biological Sciences & International Forensic Research Institute, Florida International University, Miami, FL, USA; c Biological Sciences, University of Chicago, Chicago, IL, USA; d Department of Early Childhood Education, Faculty of Arts and Education, University of Stavanger, Stavanger, Norway

**Keywords:** Forensic science, forensic entomology, Calliphoridae, *Lucilia caesar*, *Lucilia illustris*, taxonomy, AFLP analysis

## Abstract

Common DNA-based species determination methods fail to distinguish some blow flies in the forensically and medically important genus *Lucilia* Robineau-Desvoidy. This is a practical problem, and it has also been interpreted as casting doubt on the validity of some morphologically defined species. An example is *Lucilia illustris* and *L. caesar*, which co-occur in Europe whilst only *L. illustris* has been collected in North America. Reports that these species shared both mitochondrial and nuclear gene sequences, along with claims that diagnostic morphological characters are difficult to interpret, were used to question their separate species status. We report here that amplified fragment length polymorphism profiles strongly support the validity of both species based on both assignment and phylogenetic analysis, and that traditional identification criteria based on male and female genital morphology are more reliable than has been claimed.

## Introduction

Flies within the forensically and medically important genus *Lucilia* Robineau-Desvoidy provide several examples of mitochondrial DNA (mtDNA) paraphyly [1]. This presents a problem for identifying larval specimens during investigation of a suspicious death or myiasis [2], although some of these problems may be overcome with detailed species distribution knowledge [3]. In the most extensively studied example of *L. cuprina* and *L. sericata*, shared haplotypes may have resulted from mtDNA introgression because of hybridization [4]. Both genetic and morphological evidence support the existence of natural hybrids [4–9]. There are several other examples in which the mtDNA barcode failed to recover monophyletic phylogenies in Calliphoridae, e.g. *Protocalliphora* spp. [10] and *Calliphora aldrichia*/*montana* [11]. Other *Lucilia* species have been investigated less than *L. sericata*/*cuprina*, but Sonet et al. [12] found that *L. caesar*/*illustris* shared both mtDNA and nuclear ribosomal genotypes, leading those authors to question the insects’ status as separate species. In contrast, the *L. caesar* and *L. illustris* examined by GilArriortua et al. [13] did not share any ribosomal sequence. Whilst morphological analysis also indicates these species are closely related [14], they are morphologically distinct and display different environmental preferences [15].

It may be that this apparent conflict between morphology and molecular systematic analysis concerning the validity of *L. caesar* and *L. illustris* reflects the small amount of the genome that has been previously characterized. Because amplified fragment length polymorphism (AFLP) profiles represent an easy and inexpensive broad sample of the entire genome [16], such data are more likely than barcodes to recover the true phylogeny for closely related taxa [10]. We report here that *L. caesar* from Europe and *L. illustris* from Europe and North America, that could not be separated using *Cytochrome c oxidase* subunit I (COI) haplotypes, were reciprocally monophyletic for AFLP genotypes and could be reliably identified by genetic assignment.

## Materials and methods

### Fly collection

All flies were collected by hand net either on the flowers of ground elder or Apiaceae, on refuse, or by using liver or fish baits, and placed immediately in absolute ethanol to kill and preserve (and stored for <4 h at 8 °C, then stored at −20 °C). Each ethanol-preserved specimen was identified using the keys in [15] for the European specimens and [17] for the North American specimens (Table 1).

**Table 1.** Collection locations, sex and dates for the specimens used in this study.

Species	Sample Identifier	Sex	Sample collection location (latitude, longitude)	Sample collection date
*Lucilia caesar*	Lc0001	F	Sagtomta, Norway (60.03834, 10.86178)	6/22/2016
	Lc0002	M	Sandermosen stasjon, Norway (59.99831, 10.79597)	6/21/2016
	Lc0003	M	Sandermosen stasjon, Norway (59.99831, 10.79597)	6/21/2016
	Lc0004	F	Sandermosen stasjon, Norway (59.99831, 10.79597)	6/21/2016
	Lc0005	F	Sandermosen stasjon, Norway (59.99831, 10.79597)	6/21/2016
	Lc0006	F	Sagtomta, Norway (60.03834, 10.86178)	6/22/2016
	Lc0007	F	Sagtomta, Norway (60.03834, 10.86178)	6/22/2016
	Lc0010	F	Sagtomta, Norway (60.03834, 10.86178)	6/22/2016
	Lc0011	F	Sagtomta, Norway (60.03834, 10.86178)	6/22/2016
	Lc0016	M	Gjerdrumveien, Norway (60.07687, 11.11750)	6/28/2016
	Lc0029	M	Renseveien ved Gardermoen, Norway (60.16913, 11.12702)	6/28/2016
	Lc0030	F	Renseveien ved Gardermoen, Norway (60.16913, 11.12702)	6/28/2016
	Lc0031	M	Renseveien ved Gardermoen, Norway (60.16913, 11.12702)	6/28/2016
	Lc0032	F	Parkeringsplassen ved Solemskogen, Norway (59.97850, 10.81807)	7/22/2016
	Lc0033	F	Parkeringsplassen ved Solemskogen, Norway (59.97850, 10.81807)	7/22/2016
	Lc0034	F	Simadalen, Norway (60.00009, 09.72702)	7/23/2016
	Lc0035	M	Simadalen, Norway (60.00009, 09.72702)	7/23/2016
	Lc0038	F	Simadalen, Norway (60.00009, 9.72702)	7/23/2016
	Lc0045	M	Norefjellstua, Norway (60.20129, 9.58415)	7/26/2016
	Lc0060	M	Storøykilen, Norway (59.89555, 10.60711)	7/28/2016
	Lc0076	M	Gjerdrumveien, Norway (60.07687, 11.11750)	7/31/2016
	Lc0077	F	Renseveien ved Gardermoen, Norway (60.16913, 11.12702)	7/31/2016
	Lc0079	M	Blikkvegen, Norway (60.18506, 11.16292)	7/31/2016
*Lucilia illustris*	Li0017	F	Gjerdrumveien, Norway (60.07687, 11.11750)	6/28/2016
	Li0019	F	Gjerdrumveien, Norway (60.07687, 11.11750)	6/28/2016
	Li0021	M	Gjerdrumveien, Norway (60.07687, 11.11750)	6/28/2016
	Li0022	M	Gjerdrumveien, Norway (60.07687, 11.11750)	6/28/2016
	Li0024	M	Gjerdrumveien, Norway (60.07687, 11.11750)	6/28/2016
	Li0027	F	Renseveien ved Gardermoen, Norway (60.16913, 11.12702)	6/28/2016
	Li0075	M	Gjerdrumveien, Norway (60.07687, 11.11750)	7/31/2016
	Li0088	F	Blikkvegen, Norway (60.18506, 11.16292)	7/31/2016
	Li0091	F	Blikkvegen, Norway (60.18506, 11.16292)	7/31/2016
	Li0113	M	Gjerdrumveien, Norway (60.07687, 11.11750)	8/16/2016
	Li0116	M	Gjerdrumveien, Norway (60.07687, 11.11750)	8/16/2016
	Li0118	F	Blikkvegen, Norway (60.18506, 11.16292)	8/16/2016
	Li0119	F	Blikkvegen, Norway (60.18506, 11.16292)	8/16/2016
	Li0121	F	Blikkvegen, Norway (60.18506, 11.16292)	8/16/2016
	Li0122	F	Blikkvegen, Norway (60.18506, 11.16292)	8/16/2016
	Li0123	F	Blikkvegen, Norway (60.18506, 11.16292)	8/16/2016
	Li0124	F	Blikkvegen, Norway (60.18506, 11.16292)	8/16/2016
	Li0129	F	Blikkvegen, Norway (60.18506, 11.16292)	8/16/2016
	Li0301	n/a	Bloomington, Indiana (39.16222, −86.529167)	9/27/2015
	Li0302	n/a	Bloomington, Indiana (39.16222, −86.529167)	9/27/2015
	Li0303	n/a	Bloomington, Indiana (39.16222, −86.529167)	9/27/2015
	Li0304	n/a	Bloomington, Indiana (39.16222, −86.529167)	9/27/2015
	Li0305	n/a	Bloomington, Indiana (39.16222, −86.529167)	9/27/2015
*Lucilia sericata*	Ls0026	M	Renseveien ved Gardermoen, Norway (60.16913, 11.12702)	6/28/2016
	Ls0039	M	Sokna center, Norway (60.24034, 9.92653)	7/26/2016
	Ls0040	M	Sokna center, Norway (60.24034, 9.92653)	7/26/2016
	Ls0041	M	Sokna center, Norway (60.24034, 9.92653)	7/26/2016
	Ls0042	M	Sokna center, Norway (60.24034, 9.92653)	7/26/2016
	Ls0043	M	Sokna center, Norway (60.24034, 9.92653)	7/26/2016
	Ls0044	M	Sokna center, Norway (60.24034, 9.92653)	7/26/2016
	Ls0046	M	Storøykilen, Norway (59.89555, 10.60711)	7/28/2016
	Ls0050	F	Storøykilen, Norway (59.89555, 10.60711)	7/28/2016
	Ls0055	F	Storøykilen, Norway (59.89555, 10.60711)	7/28/2016
	Ls0056	M	Storøykilen, Norway (59.89555, 10.60711)	7/28/2016

### DNA extraction, genetic and phylogenetic analysis

DNA was extracted from the head of each fly, with the remaining portion of each specimen placed back in ethanol and kept at −20 °C. The DNA was extracted using manufacturer's protocols for the Qiagen DNeasy Blood and Tissue Kit (Qiagen, Valencia, CA, USA), with a final elution volume of 75 µL. DNA extracts were stored at −20 °C until further use.

For the generation of mtDNA haplotypes, the 5’ end of the *COI* gene was amplified using a Promega 2× PCR master mix (Promega Corp, Madison, WI, USA). The sequences of the primers (TY-J-1460/C1-N-1840) purchased from IDT (Integrated DNA Technologies, San Jose, CA, USA) and thermal cycler program are described in [18]. PCR product was visualized using a 1.5% agarose gel stained with SYBR® Safe (Thermo Fisher, Waltham, MA, USA) under UV light. PCR product was cleaned with a QIAquick PCR Purification Kit (Qiagen), and sent to a commercial DNA sequencing service (Macrogen, Seoul, Republic of Korea) for sequencing of both strands by standard sequencing using capillary electrophoresis. Sequence data files were edited and aligned with Sequencher software (Soft Genetics, State College, PA, USA). All specimens produced the complete 372-base sequence corresponding to positions 1-372 in L14946 [2]. Because one resulting haplotype was shared between *L. illustris* and *L. caesar*, we performed no phylogenetic analysis of mtDNA sequence data.

For the generation of AFLP profiles, the methods outlined in [19] were used, using the corrected adaptor and primer sequences from [20]. Each selective PCR product was separated and detected using a 3500 Genetic Analyser (Life Technologies, Carlsbad, CA, USA) with 0.3 µL Genescan LIZ 600 size standard (Life Technologies), 1 µL PCR product and 9 µL HiDi formamide (Life Technologies). Data were analysed using GeneMarker (Softgenetics) for 1 bp bin sizes, and exported to Excel (Microsoft Corp., Redmond, WA, USA). Data were then objectively sorted for loci with >4 alleles present in the total sample, all other alleles were eliminated. The final, combined data-set containing all the loci from all four selective PCR amplifications were analysed using AFLPop [21] for assignment using a minimum log-likelihood difference of 3 as in [22]. The AFLP data were also used for maximum parsimony analysis [23], rooted using *L. sericata* as the outgroup, with 1000 bootstrap replicates.

## Results

Sequence analysis produced 11 haplotypes (Table 2). The haplotype that was most commonly observed for *L. illustris* (18 specimens, including individuals from Europe and N. America) was also found in one *L. caesar* (Lc0001), therefore our specimens could not be separated based on the small region of the *COI* gene.

**Table 2.** Distribution of distinct Lucilia spp. cytochrome oxidase one haplotypes observed in this study.

Haplotype designation	Specimens^a^	Accession numbers
1	Lc0001, Li0019, Li0021, Li0022, Li0027, Li0075, Li0088, Li0091, Li0116, Li0118, Li0122, Li0123, Li0124, Li0129, Li0301, Li0302, Li0303, Li0304, Li0305	*L. caesar* K778682 *L. illustris* K778683
2	Li0113	K778684
3	Li0119, Li0121	K778685
4	Li0017, Li0024	K778686
5	Lc0002, Lc0004, Lc0005, Lc0007, Lc0016, Lc0029, Lc0031, Lc0032, Lc0045, Lc0060, Lc0076	K778687
6	Lc0006, Lc0034, Lc0077	K778688
7	Lc0003, Lc0010, Lc0030, Lc0033, Lc0035, Lc0038, Lc0079	K778689
8	Lc0011	K778690
9	Ls0026, Ls0039, Ls0041, Ls0043, Ls0044, Ls0050, Ls0055, Ls0056	K778691
10	Ls0040	K778692
11	Ls0042, Ls0046	K778693

a Specimen codes correspond to those in Table 1.

**Figure 1.**
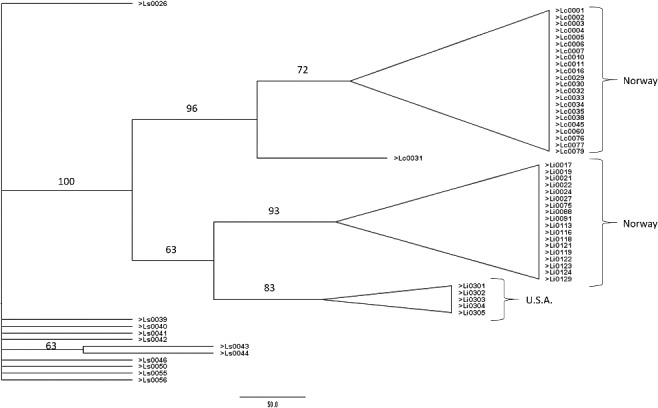
Maximum parsimony (MP) bootstrap consensus tree of *Lucilia caesar* (Lc), *L. illustris* (Li), and *L. sericata* (Ls) amplified fragment length polymorphism (AFLP) genotypes. See Table 1 for specimen information.

A total of 323 AFLP loci for 57 specimens were generated using four selective primers in three *Lucilia* species ranging in size between 100 and 500bp. Assignment tests were done using the leave-one-out procedure for allocation, an individual sample was removed from the data, frequencies are re-calculated, and then the “unknown” was allocated to a population if the likelihood was 1000 times more likely to belong to that group. Two different assignment tests were done, one in which all of the *L. illustris* were considered a single group (three groups total), and a second one in which the *L. illustris* were split into two groups (North American and European, four groups total). In both cases, 100% of the specimens allocated to the correct species (not shown) or population, although the single Indiana sample is insufficient for a test of geographic structure [19,22]. The phylogenetic analysis produced very similar results (Figure 1).

## Discussion

It is common to use DNA for difficult-to-identify species identification, however, caution should be exercised with many of the *Lucilia* species if using portions of the *COI* gene [1]. This is particularly important in a forensic context, as the species, though closely related, may exhibit different developmental rates. In the case described here, the practical implications of confusing *L. illustris* and *L. caesar* are unclear because so little has been published about the development rate of *L. caesar*. That said, the limited data available suggest that mistaking one of these species for the other could result in an estimate of age that was wrong by about 10% [24]. Also, as noted earlier, these two species prefer different ecological habitats [15], so it is conceivable that an investigator might incorrectly conclude that the corpse had, or had not, been moved. In addition, we believe that correct specimen identification during forensic analysis constitutes best practice under any circumstances. It reflects on the analyst's competence, and even if not important in the present investigation one never knows if it will be important for a future re-examination of the case.

Our molecular systematic analysis supports separate species status for *L. illustris* and *L. caesar* despite the fact that, as was reported by other authors [12,13], they could not be distinguished based on mtDNA. However, given that the results of similar nuclear gene sequence analyses depended on the geographic source of the specimens [12,13], we believe that AFLP surveys of other parts of these species’ distribution, e.g. East Asia, are warranted.

Although this is a molecular systematics analysis, we wish to affirm our confidence in the traditional morphological approach to taxonomy. We think that limited genotype data, such as are usually produced in an effort to develop a species-diagnostic test, are less compelling compared to traditional methods of alpha taxonomy. Whilst *L. illustris* is the only species in the genus in North America with small black setulae on the subcostal sclerite, there are three species in Europe with such setulae [15]. One of them (*L. ampullacea*) lacks a coxopleural streak, whilst this structure is present in the two others, *L. caesar* and *L. illustris*. These two species are easily separated in the male sex to the extent that we believe few would argue against their status as valid species [15]. Because, in our opinion [12], misinterpreted [15] to support claims such as “dried female [*L. illustris* and *L. caesar*] specimens … cannot be accurately identified by morphology”, or that the shape of the female tergite VI is an unreliable character for distinguishing these two species, we will discuss these structures in detail. The shape of tergite VI as a means to separate females of *L. caesar* and *L. illustris* was first introduced by Spence [25] for the British fauna, and subsequently used by Zumpt [26] for his monograph of the Palaearctic Calliphoridae. The shape and vestiture of tergite VI in females are very distinctive when in flat ovipositor slide preparations. The essential features of tergite VI for separating *L. caesar* and *L. illustris* are as follows: (1) the dorsal margin of tergite VI convex in profile, *vs.* straight; (2) the distal margin in profile with small setae in upper and lower part, with a long section without or almost without small setulae in between, vs. full uninterrupted row of strong marginal setae; (3) the distal margin in profile long (high) and at right angles to the dorsal margin, vs. short and forming a more acute angle with the dorsal margin. In addition, sternite VIII in the ovipositor is strikingly long in *L.caesar*, almost covering the hypoproct from below ([27] p. 223, figs. 13y and 14y). These features are often directly observable in dried female specimens ([28] figs. 3n and 3p), and if not are readily revealed in KOH-treated and subsequently dissected specimens. The features are also easily examined in ethanol preserved material, if necessary by removing parts of tergite V to expose tergite VI and the tip of the ovipositor. The male genitalia, similarly, differ greatly in the shape of the cerci and surstyli, and the size of the epandrium [15], also as easily examined in dried as well as ethanol preserved specimens. In our analysis, all of the females grouped with the correct males, showing that there is no problem with their identification and association with conspecific males.

We disagree with the suggestion that the morphological differences between the males are “subtle”, and that the “currently assumed interspecific differentiation between *L. caesar* and *L. illustris* may merely represent intraspecific variation” [12].

## Supplementary Material

AFLP_Data.xlsx
